# Urbanization and Habitat Diversity Promote Endozoochorous Seed Dispersal by Raccoon Dogs Within Forest Fragments in Tokyo

**DOI:** 10.1002/ece3.72516

**Published:** 2025-11-14

**Authors:** Harsh Yadav, Yuki Iwachido, Shyam S. Phartyal, Takehiro Sasaki

**Affiliations:** ^1^ Graduate School of Environment and Information Sciences Yokohama National University Yokohama Kanagawa Japan; ^2^ Department of Forestry Mizoram University Aizawl India; ^3^ Institute for Multidisciplinary Sciences Yokohama National University Yokohama Kanagawa Japan

**Keywords:** endozoochory, habitat diversity, *Nyctereutes procyonoides*, seed dispersal, urbanization

## Abstract

Urbanization affects biodiversity and essential ecological functions, such as animal‐mediated seed dispersal. Novel urban habitats introduce environmental heterogeneity that alters the effectiveness of animal‐mediated seed dispersal. Despite growing urbanization, the additive effect of urban indicators on habitat diversity in shaping animal‐mediated seed dispersal by urban mammals remains poorly understood. Hence, this study examined the influence of urban indicators and habitat diversity on endozoochorous seed dispersal by urban‐adapted raccoon dogs within urban forest fragments of the Tokyo metropolitan area. We surveyed raccoon dog latrines across 19 urban green spaces and identified 66 plant species in their feces with most of them primarily native. The most frequently dispersed species were *Morus australis* and *Aphananthe aspera*. However, discrepancies between plant species found in the feces and those present in local vegetation suggest a broader foraging range beyond the study sites. The results showed that the presence of more built‐up area, significantly increased plant species richness in feces. The additive effect of built‐up areas and habitat diversity increased endozoochory at larger scales. This underscores the role of seed dispersers in maintaining plant diversity in urban forest fragments, where increasing habitat diversity at large scales supports more plant dispersal. Our findings highlight the importance of native plants in endozoochory, which supports their conservation by dispersing them. This study reinforces the ecological importance of urban‐adapted raccoon dogs in maintaining biodiversity and ecological processes despite the challenges posed by urbanization.

## Graphical abstract

A study of raccoon dogs in Japan's urban forests revealed they are vital seed dispersers, primarily for native plant species. Notably, the dispersed plant species richness increased significantly with respect to habitat diversity and built‐up area compared to other urbanization indicators. This finding underscores the crucial ecological role of urban‐adapted wildlife in maintaining plant biodiversity within urban forest fragments.

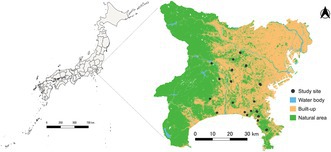

## 1 Introduction

Animal‐mediated seed dispersal (zoochory) is an important ecological function that facilitates plant movement and establishment, thereby making it indispensable for ecosystem maintenance (Herrera 2002; Corlett 2017). Endozoochory, a type of zoochory, occurs when animals ingest fruits or seeds and later disperse them via defecation (Herrera 2002; Yadav and Phartyal 2023). Many plant species depend on endozoochory for seed dispersal and have co‐evolved to enhance mutualistic seed dispersal interactions (Corlett 2017; Enomoto et al. 2018; Takatsuki et al. 2018; Stanley and Arceo‐Gómez 2020).

The impact of anthropogenic pressures on plants has been extensively studied (Rega‐Brodsky et al. 2022). Previous studies have shown that anthropogenic pressures such as urbanization lead to species invasion, with biotic homogenization recognized as a prevalent phenomenon (McKinney 2006). However, studies have also highlighted the positive role of urban green spaces in maintaining native plants through dispersal (Staude 2024a). For instance, waterfowl birds have been dispersing native plants in the urban habitats (Tóth et al. 2023). The urban‐adapted corvids are active dispersers of native plants in urban areas (Green et al. 2019). The cultivation and plantation of native plants in urban green spaces can contribute to their conservation when dispersed by animals interacting with such plants having favorable traits (Staude 2024a, 2024b). Despite the evidence of native plant dispersal by animals in urban areas, urbanization can affect ecological processes, such as endozoochory, by reducing functional diversity, facilitating the spread of non‐native species, or creating unsuitable conditions for seed deposition (Markl et al. 2012; Stanley and Arceo‐Gómez 2020; Yadav et al. 2024, 2025). These effects may be exacerbated by rising urban indicators, such as human population density, artificial light, and impervious surfaces (United Nations (UN) 2018; United States Environment Protection Agency 2024). Despite the importance of these factors, recent reviews have highlighted the limited research on endozoochorous seed dispersal in urban ecosystems, with the effects of urbanization rarely considered in research on urban endozoochory (Yadav et al. 2025). In this regard, understanding the effects of various urban indicators on ecological interactions, especially endozoochory, is crucial and currently remains largely unexplored.

The global population is projected to reach 9 billion by 2050, with approximately 67% of people likely to reside in urban areas (United Nations (UN) 2018). The consequences of this shift include ecosystem degradation, natural habitat fragmentation, and novel habitat formation (Theodorou 2022; Iwachido et al. 2023; Pineda‐Pinto et al. 2024). Natural habitat diversity may vary based on the spatial scale and complementary effects of built‐up areas, such as increasing accessibility, which can influence animal diets (Kauhala and Ihalainen 2014; Nottingham et al. 2024). Studies have shown that urbanization leads to an increase in spatial heterogeneity creating diverse habitat types (Aronson et al. 2017; Zhou et al. 2017; Iwachido et al. 2024). A positive relationship between habitat diversity and species diversity was seen by providing more niche space and facilitating the coexistence of species (Tews et al. 2004; Hortal et al. 2009). However, several studies have also shown insignificant or negative effects of heterogeneity (Laanisto et al. 2013; Gazol et al. 2013). For instance, the area‐heterogeneity trade‐off hypothesis suggests that compositional heterogeneity or habitat diversity may not necessarily promote species richness (Chiron et al. 2024). Similarly, these effects can be further exacerbated for ecological processes, such as endozoochorous seed dispersal, in urban conditions when built‐up areas have an additive influence on habitat diversity (Zepeda et al. 2025). Additionally, habitat diversity variations can modify animal‐mediated seed dispersal by urban‐adapted species such as endozoochory owing to changes in habitat preferences and resource availability (Nield et al. 2020; Stanley and Arceo‐Gómez 2020). Nevertheless, understanding their contribution to green spaces in the framework of urbanization is essential. In this context, research on urban endozoochory has rarely evaluated the effects of habitat diversity on urban‐adapted seed dispersers at varying scales.

Raccoon dogs, omnivorous members of the Canidae family, are well adapted to urban areas in East Asia (Enomoto et al. 2018). These medium‐sized animals, weighing between 4 and 10 kg (Carr 2004), also serve as seed dispersers in urban ecosystems supporting the maintenance of plant communities (Enomoto et al. 2018; Takatsuki et al. 2018). Studies have shown dispersal of fleshy and dry fruits of large trees by raccoon dogs seed dispersal and infrequent tree visitation frequency under the presence of ungulates such as wild boar (Koike and Masaki 2019; Osugi et al. 2019). Previous research also presented the undamaged seeds in frugivores such as raccoon dogs where large size seeds have better survival rates (Koike et al. 2008; Osugi et al. 2020) In addition, the ability of raccoon dogs to disperse seeds faraway from the parent plant (> 100 m) to other habitats makes them efficient seed dispersers (Sakamoto and Takatsuki 2015; Tsunamoto et al. 2020). Research also suggests that habitat diversity can support the omnivorous diet of mammals, such as that of the raccoon dog (Kauhala and Ihalainen 2014). Despite advancements in seed dispersal research related to raccoon dogs and the significance of urban endozoochory, the impact of urban indicators and habitat diversity on this seed dispersal mechanism by urban‐adapted dispersers remains largely unexplored.

In this study, we aimed to evaluate the key urban indicators influencing raccoon‐dog‐mediated seed dispersal and assess its relationship with habitat diversity. We tested the hypothesis that even though urban‐adapted animals are efficient dispersers, increasing built‐up areas may reduce the positive effects of habitat diversity by limiting the availability of natural areas. Therefore, we examined plant species richness in raccoon dog feces within urban green spaces and investigated the relationship between dispersed plant species and urbanization. Specifically, we sought to answer the following questions: (1) Do raccoon dogs primarily disperse native or non‐native plant species in urban areas? (2) How do the plant species in raccoon dog feces compare to those of local vegetation? (3) Which urban indicator has the most substantial impact on raccoon‐dog‐mediated endozoochory? (4) Does urbanization modify the effect of habitat diversity on the dispersal potential of raccoon dogs?

## 2 Methods

### 2.1 Study Area

**FIGURE 1:**
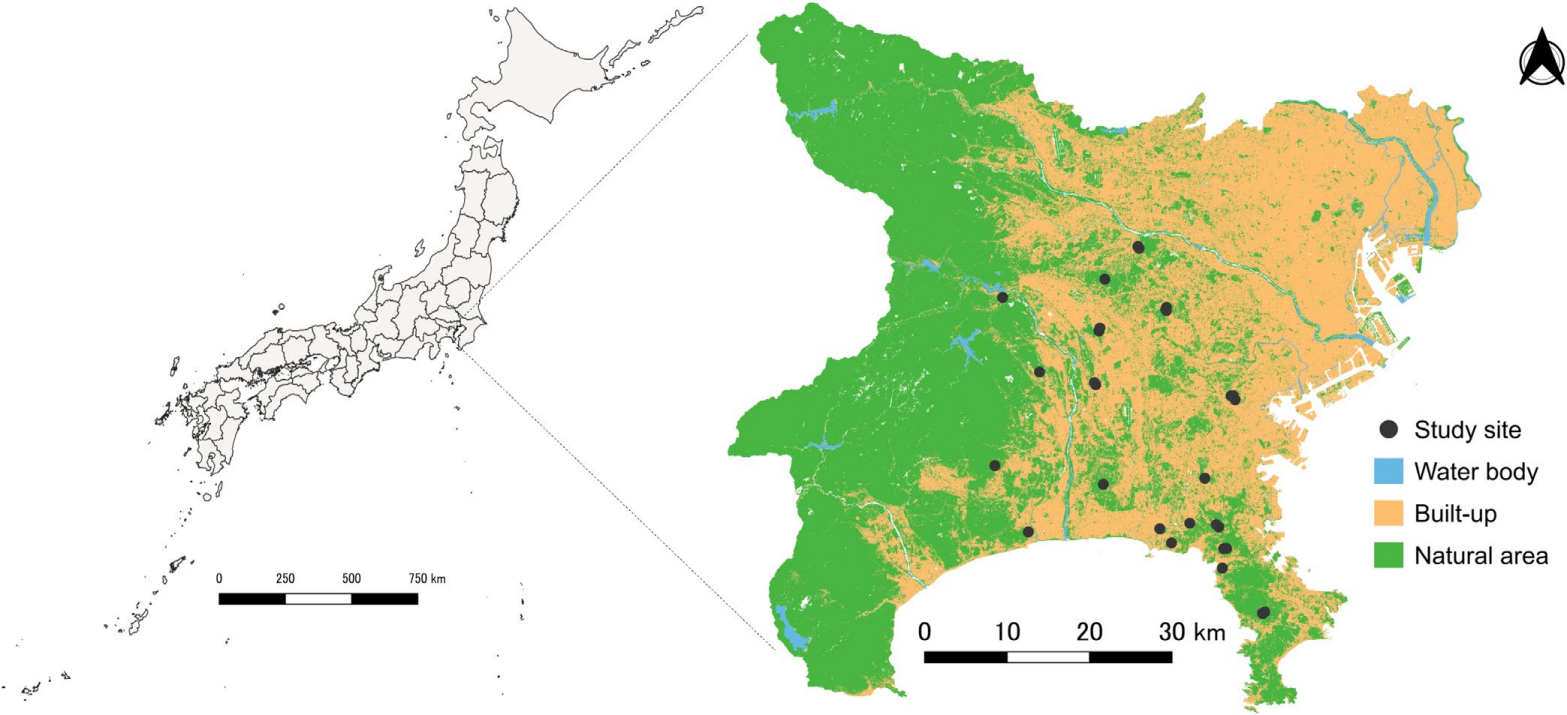
Study sites in Kanagawa and Tokyo prefectures marked with black circular points. Some of the study points overlap due to the scale of the map.

The study was conducted in urban green spaces across Kanagawa and Tokyo prefectures in Japan (Figure 1). These areas are part of the Tokyo metropolitan region, the largest global urban agglomeration (United Nations (UN) 2018). Tokyo and Kanagawa, the two most populous prefectures in Japan, have populations of approximately 14 million and 9.2 million, respectively (Ministry of Internal Affairs and Communications 2024). The climate is primarily temperate, with an average temperature of 15.8°C. The rainy season extends from early June to mid‐July, with an average annual precipitation of 1598 mm (Japan Meteorological Agency 2020; Iwachido et al. 2023). Additionally, the monthly precipitation peaks in September are due to tropical cyclones (Japan Meteorological Agency 2020). Built‐up areas across both prefectures cover 39.11% of the total land area, as estimated from land cover data (Japanese Aerospace Exploration Agency (JAXA) 2024). Since 1955–1972, rapid population growth and economic expansion have led to the increased fragmentation of green spaces in the region. These green spaces include urban forest fragments and artificial urban parks, which exhibit varying levels of human intervention, such as increasing impervious cover and introducing non‐native plant species (Iwachido et al. 2023).

### 2.2 Feces Collection and Processing

We selected 19 urban green spaces with varying levels of urbanization for raccoon dog feces collection (Figure 1, Table S2). Field surveys were conducted at all sites once per year for two consecutive years (May–July in 2023 and 2024). At each study site, two 200 × 200 m plots were established and surveyed using a zigzag line‐transect approach to ensure comprehensive coverage (Figure S1). Additionally, we established 10 subplots (2 × 2 m each, total = 20 per site) within each main plot to record the saplings of the plant species present at the sites.

All identified latrines were geotagged, and fresh fecal samples were collected. This study followed the criteria of collecting all the available feces in the latrine. The samples were washed with tap water using a series of sieves (500–212 μm). After washing, the retained seeds were air‐dried and counted to determine seed number and plant species richness. Plant species were identified using literature (Suzuki et al. 2018) and the *Seed Image Database* of Okayama University (https://www.rib.okayama‐u.ac.jp/wild/index.html). Furthermore, their nativeness was determined using *Plants of the World Online* (POWO 2024).

Seed viability was assessed using the conventional water submergence and cut‐test methods. Seeds that remained submerged were considered potentially viable, whereas floating seeds were presumed damaged or empty. Furthermore, the submerged seeds were subjected to a cut test to validate their viability. Seeds containing all the essential tissues required for germination, such as a firm, uniform, and opaque endosperm with a healthy embryo, were classified as viable. In this study, all the submerged seeds had intact endosperm and embryo. Hence, no submerged seeds were discarded after the complete seed viability test.

To understand the plant species more important in the feeding habit of raccoon dogs, we calculated the frequency of occurrence (Equation 1) and relative importance (Equation 2) of plant species found in the feces:

(1) $$\frac{\text{Species occurrence in the feces}\;\mathrm{at}\;a\;\text{site}}{\text{Total occurrence of that species across the sites}}\times 100$$

(2) $$\frac{\text{Frequency of occurrence}}{\mathrm{Sum}\;\text{of}\;\mathrm{all}\;\text{occurrences across the sites}}\times 100$$

### 2.3 Urbanization Data and Habitat Diversity

We used these three indicators of urbanization, namely built‐up area, artificial light, and population density, recognized for being detrimental to various ecological processes, such as species interactions (Chen et al. 2023). Built‐up area (hereafter urbanization rate) data was extracted from the (Japanese Aerospace Exploration Agency (JAXA) 2024) and artificial light data from the New World Atlas of Artificial Night‐Sky Brightness (Falchi et al. 2016). Population density data were retrieved from the Centre for Socioeconomic Data and Applications of the National Aeronautics and Space Administration (Center for International Earth Science Information Network (CIESIN), Columbia University 2018) (Table S3). The urbanization rate was the proportion of built‐up area in the respective buffer category.

The data layers for urban indicators were entered in the QGIS software (version 3.34.11). We created a 200‐m buffer around the raccoon dog latrine point and subsequently increased it incrementally by 200 m up to 2000 m. Finally, urbanization indices were extracted for the 10 buffer categories (200–2000 m) using zonal histogram analysis in QGIS software (version 3.34.11). The zonal histogram analysis gives the proportion of the urban indicator in the respective buffer category. Because population density data were available in grid format, the zonal statistics tool was used to extract the mean values within the buffer area. Furthermore, we used the land cover data (Japanese Aerospace Exploration Agency (JAXA) 2024) to measure habitat diversity. The land cover data comprised 11 categories other than built‐up area. We removed water body and bare ground from the available habitat categories and performed zonal histogram analysis to get the proportion of remaining habitats in the respective buffer category.

### 2.4 Data Analyses

All analyses were conducted using the R version 4.3.3 (R Core Team 2024). To evaluate whether native or non‐native species were deposited in the urban forest fragments, we used the plotweb function from the bipartite package (Dormann et al. 2008) to visualize site‐specific species found in the feces. A Wilcoxon rank‐sum test from the stats package was performed to examine the difference between the native species and plant species in feces across the sites. An additional Wilcoxon rank‐sum test compared plant species richness in feces and that surveyed in the subplots to assess the contribution of raccoon‐dog‐mediated seed dispersal. Urbanization indices were scaled using the scale function. To test the effect of urban indicators and habitat diversity on the number of dispersed plant species, we performed a generalized linear mixed model analysis using the lme4 package (Bates et al. 2015) with a Poisson distribution in all models. The number of species within the feces was used as the response variable, while the study site was used as the random effect.

In the first set of analyses, separate models were created to determine relevant urban indicators for habitat diversity analysis, with each urban indicator in its respective buffer category serving as an explanatory variable. The most parsimonious model with respect to the urban indicator was checked based on Akaike information criterion values.

We used the diversity function of the vegan package (Oksanen et al. 2022) and the Shannon index to examine the habitat diversity for each buffer radius based on land cover categories.

No correlation was observed between the selected urban indicator and Shannon index. In the second set of analyses, the most significant urban indicator from the buffer category was included alongside the Shannon index as an additional explanatory variable. Model accuracy was assessed using the simulateResiduals function from the DHARMa package (Hartig 2024). Furthermore, the testDispersion and testZeroInflation functions were applied to evaluate model robustness. The results were visualized using the ggplot2 package (Wickham 2016).

## 3 Results

We collected 66 raccoon dog fecal samples that comprised 66 plant species (2382 seeds). Among the identified plant species, 10 were identified only at the genus level, while nine remained unidentified. The feces contained plant species belonging to 40 families and 51 genera, with Rosaceae (*n* = 7 species) and *Prunus* (*n* = 4) being the most dominant family and genus, respectively. The most frequently occurring plant species in feces across sites were *Aphananthe aspera*, *Morus australis*, *Aucuba japonica* , *Celtis sinensis* , and *Cornus controversa* . Conversely, *M. australis* (*n* = 618 seeds), *Prunus speciosa* (*n* = 349), and *Cornus controversa* (*n* = 248) had the greatest number of seeds (Table S1).

Among the 47 identified plants at the species level, 85% (*n* = 40 species) were native (Figure S2, Table S2). The seed viability assessment showed that 88.87% of the seeds were intact and viable (Figure S3, Table S2). The most dispersed species were trees (*n* = 28), followed by shrubs (*n* = 8) and herbs (*n* = 8). On average, 11.84 ± 5.89 species were found in the feces across the sites (ranging from 1 to 25). Conversely, 125.36 ± 116.6 seeds were present in the feces (ranging from 9 to 494).

**FIGURE 2:**
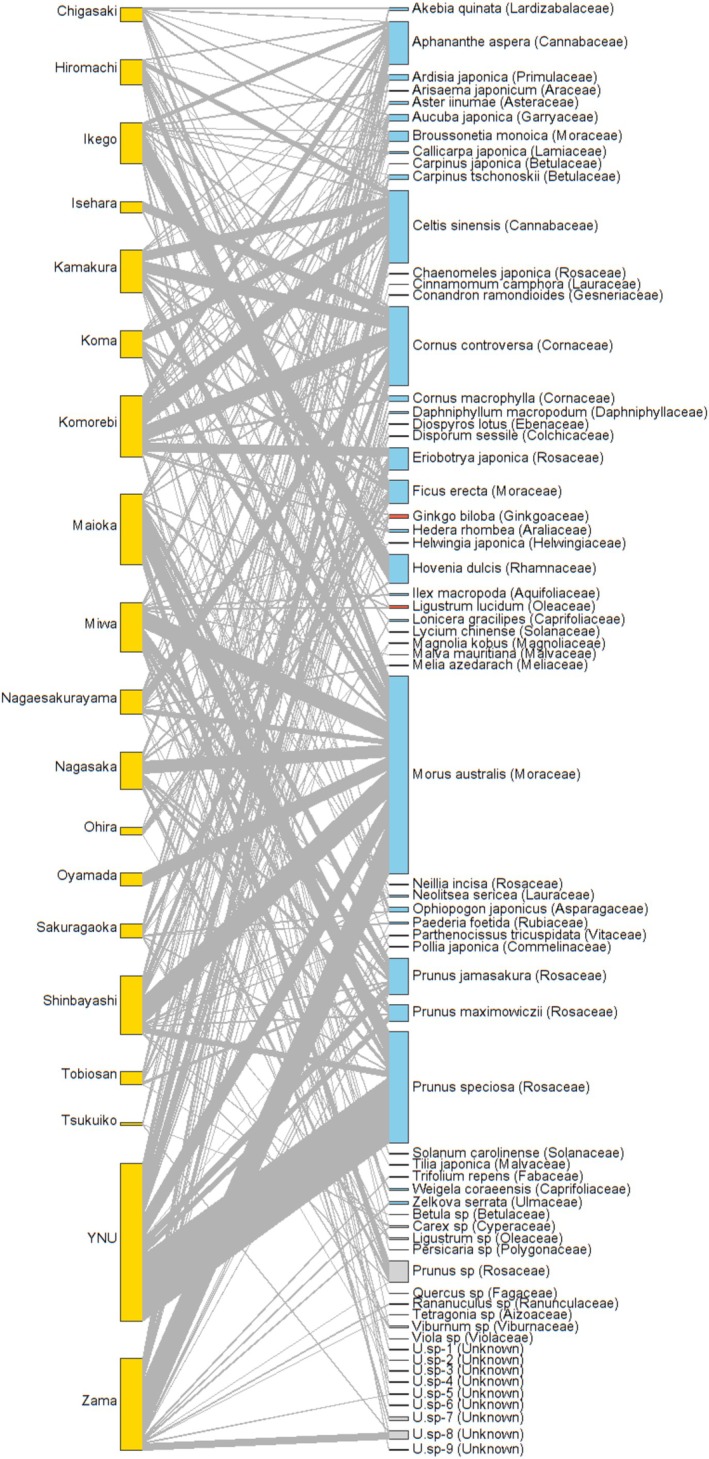
Plant species present in the feces of *Nyctereutes procyonoides* at each site. The left side denotes the study sites, while the right side indicates the plant species along with family names (in the parentheses). The thicknesses of the interacting nodes represent the number of seeds at each site. Colors on the plant species bars: Blue = native species, orange = non‐native species, and gray = unknown. U.sp‐1 to U.sp‐9 are unknown species.

Among all species, *A. aspera* and *M. australis* were the most frequent in the feeding habits of raccoon dogs because they were present in the feces at most sites (Figure 2). Both species exhibited the highest relative importance (30.41%) (Table S1). Interestingly, both species had different seed sizes. Twenty‐six species were detected exclusively in feces only at individual sites and were absent in samples from other sites. No significant difference was found between the total dispersed and native species found in the feces across the sites (*W* = 1.34, *p* = 0.17). However, a significant difference was observed between plant species present in the feces and those inhabiting the site (*W* = 4.89, *p* = 0.005) (Figure S4).

The assessment of the three urban indicators on the number of species found in the feces revealed a positive influence of these indicators. However, built‐up area (urbanization rate) was the most significant indicator positively influencing plant species richness in raccoon dog feces across buffer categories. Additionally, the built‐up area of the 1200 m buffer category was the most effective across the urban indicator analysis (estimate = 0.021, *p* < 0.001) (Table S4).

**FIGURE 3:**
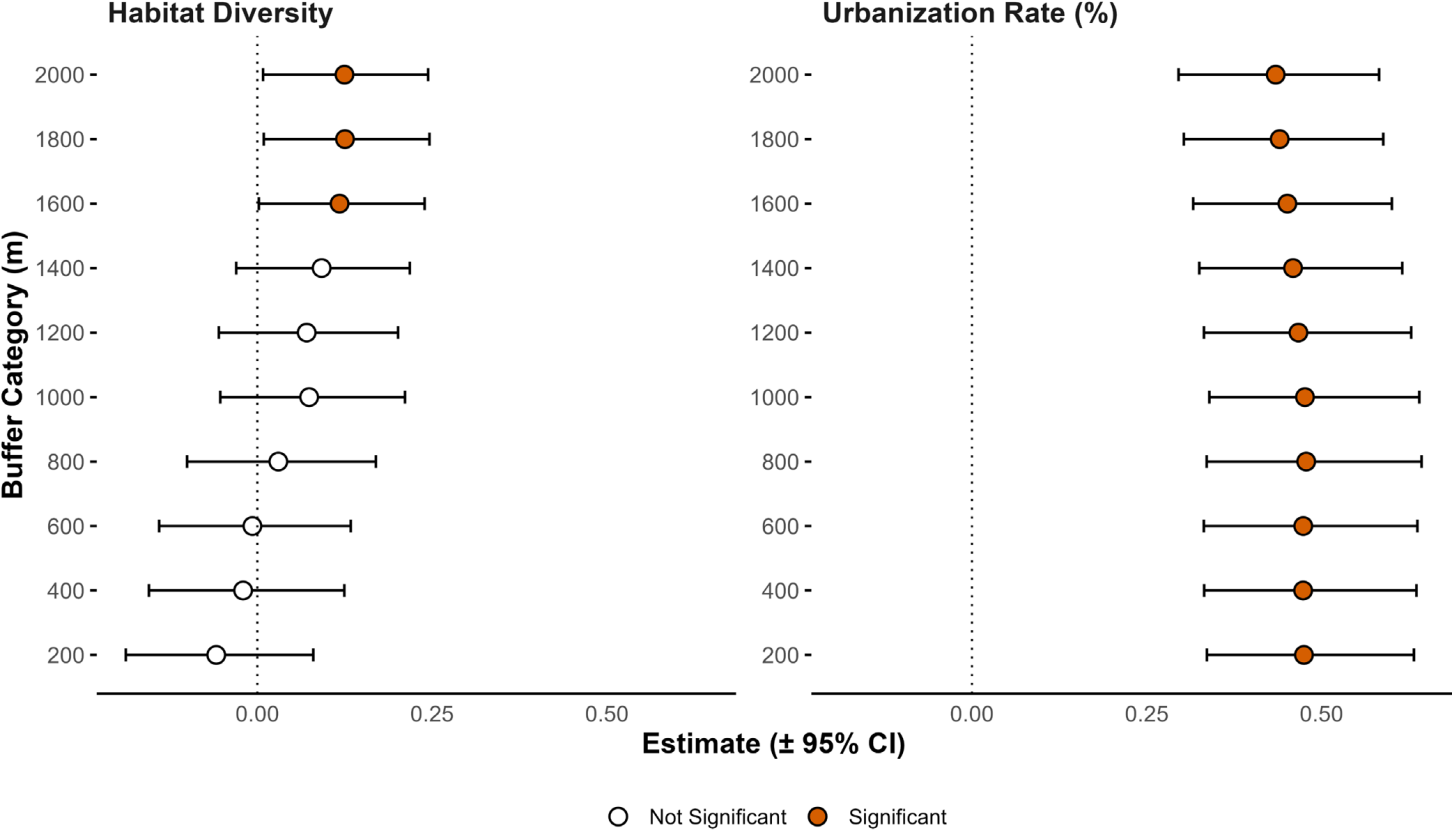
Effects of habitat diversity and urbanization rate on plant species richness. The left side denotes the estimates of habitat diversity, while the right side indicates the estimates of urbanization rate within respective buffer radius categories. The colored points represent significant effects, whereas the tails indicate confidence intervals.

When incorporating the built‐up area (urbanization rate) of the 1200 m buffer category as an additional explanatory variable along with the Shannon index, significant effects were observed at larger buffer scales. The 1600 m buffer (urbanization rate: estimate = 0.45, *p* < 0.001; Shannon index: estimate = 0.12, *p* = 0.04), 1800 m buffer (urbanization rate: estimate = 0.44, *p* < 0.001; Shannon index: estimate = 0.125, *p* = 0.033), and 2000 m buffer (urbanization rate: estimate = 0.43, *p* < 0.001; Shannon index: estimate = 0.124, *p* = 0.03) significantly influenced the plant species dispersed by raccoon dogs (Figure 3).

Although insignificant, a notable negative effect of habitat diversity was observed at smaller buffer scales (200–600 m) when the urbanization rate was included as an explanatory variable. However, no significant effect of habitat diversity on the number of seeds was detected across the various buffer scales (Figure S5).

## 4 Discussion

Urbanization has been widely recognized for its negative effects on species diversity, often leading to biotic homogenization (Olden et al. 2006). However, this does not necessarily translate to reduced ecological interactions, such as endozoochorous seed dispersal. In this study, we critically and empirically assessed the role of raccoon dogs as endozoochorous seed dispersers in urban environments, revealing that urban indicators positively supported this seed dispersal mechanism. Among these indicators, the urbanization rate (proportion of built‐up area) had the most significant effect, potentially due to the increased availability of fleshy‐fruited plants in urban environments, which attracted urban‐adapted dispersers and provided greater accessibility to feeding resources in built‐up areas (Stanley and Arceo‐Gómez 2020; Yadav et al. 2025). However, other urbanization‐related factors, such as habitat diversity, are also likely to influence endozoochory in urban areas.

Raccoon dogs are highly adapted to urban ecosystems and consume various plant species with different seed sizes, making them important seed dispersers in urban areas (Osugi et al. 2020). The high relative importance of *M. australis* (seed mass = 1.36 g) and *A. aspera* (seed mass = 110 g) in the diet of raccoon dogs underscores their ability to ingest and disperse seeds of various sizes (Society for Ecological Restoration, International Network for Seed Based Restoration and Royal Botanic Gardens Kew 2023; Tabata and Morimoto 2017). Their ability to transport plant species with a broad spectrum of seed sizes enhances endozoochorous seed dispersal and contributes to plant diversity in urban landscapes. Omnivorous species typically exhibit extended gut passage times for seeds, enabling long‐distance dispersal (Stanley and Arceo‐Gómez 2020). Previous studies have shown that urban‐adapted dispersers interact with a diverse range of plant species, thereby increasing the likelihood of novel interactions (Schneiberg et al. 2020; Stanley and Arceo‐Gómez 2020). This phenomenon may arise from their generalist foraging behavior and shifts in species interactions, wherein urban‐adapted species replace locally extinct specialists (Schleuning et al. 2020).

Contrary to the common assumption that urbanization promotes the seed dispersal of non‐native plant species due to homogenized disperser communities (McKinney 2006; Schneiberg et al. 2020), our findings highlight the role of raccoon dogs in dispersing predominantly native plant species to various habitats in urbanized areas, thereby contributing to urban plant diversity. Moreover, the mismatch between the plant species present in the feces and those present at the study sites suggests that raccoon dogs may forage beyond the study area by taking advantage of the diverse habitats available. In addition, the same activity also allows raccoon dogs to disperse the seeds across habitats. One reason could be the plantation of fruit‐rich native plants in gardens, artificial parks, and other urban green spaces attracting raccoon dogs beyond natural forest fragments when searching for food, thereby facilitating the dispersal of native plants to new locations (Staude 2024a, 2024b; Kauhala and Ihalainen 2014; Stanley and Arceo‐Gómez 2020).

Our investigation into the effects of different urban indicators revealed a significant relationship with the urbanization rate, contributing to the growing body of literature on the role of human society in shaping urban ecosystems (Grimm et al. 2008; Gelmi‐Candusso and Hämäläinen 2019). Previous studies have reported the predominantly adverse effects of infrastructure and human presence on species (Lasky and Bombaci 2023). However, other studies have suggested that a higher human population density can positively impact wildlife survival (Zepeda et al. 2025). Our findings support this perspective, demonstrating that raccoon dogs benefit from urban indicators. Nonetheless, the long‐term effects of urbanization on these urban‐adapted species should be considered to better understand their ecological functions.

The relationship between urbanization and habitat diversity in terms of endozoochory by raccoon dogs has not been extensively explored in previous studies. Rodriguez‐Perez et al. (2012) emphasized the importance of disperser behavior in seed dispersal dynamics across different habitat types and spatial scales. A similar pattern emerged in our study, where large spatial‐scale buffers (1600–2000 m) significantly influenced the plant species detected in raccoon dog feces due to the presence of a wide range of habitats allowing them to have a diverse feeding breadth. Furthermore, the positive influence of habitat diversity on plant species in feces suggests that the presence of diverse habitats in more urbanized areas could support urban‐adapted animals to disperse more plant species. This finding underscores the importance of natural habitat diversity in shaping disperser behavior in urban landscapes. For instance, reduced availability of natural habitats may drive raccoon dogs to intensively explore built‐up areas with low availability of fruited plants.

Our findings highlight that natural habitat diversity plays a key role in enhancing seed dispersal services provided by raccoon dogs. However, a notable negative effect of habitat diversity at smaller scales, when the urbanization rate is incorporated, may underscore the impact of urbanization when habitat diversity is limited. The high accessibility and abundant food resources in natural habitats within urban ecosystems result in smaller home ranges for raccoon dogs compared to those in rural areas in Japan (Mitsuhashi et al. 2018). This factor may contribute to the slight negative pattern observed at small spatial scales, where limited habitat diversity coupled with built‐up areas can be detrimental. Nevertheless, our findings suggest that urbanization facilitates habitat diversity at larger scales, contradicting initial assumptions that increased built‐up areas may adversely impact raccoon‐dog‐mediated endozoochory. However, further research is needed to explore habitat diversity at small scales and potential urbanization effects on urban endozoochory. This underscores the urgent need for urban planning and management strategies that support ecological processes.

Our study did not assess the fate of dispersed seeds, particularly their germination potential and survival rates. Additionally, we did not account for the effects of human food provisioning on animals (Sengupta et al. 2022), as our primary objective was to examine the linkages between urban indicators, habitat diversity, and seed dispersal. Furthermore, the study did not account for the inflow of feces in the latrine that could also be dependent on the population density of raccoon dogs in the area studied. The seeds available in the latrine are also potentially driven by the decomposition rate of feces depending on the local environmental conditions. In this study, seeds of plants fruiting in different seasons were found in the feces at the latrines. These latrines act as communal spots of feces defecation used by multiple raccoon dogs. One possibility could be the constant use of latrines by multiple raccoon dogs for longer periods without external interference and dumping seeds from several locations. In addition, favorable conditions such as slow decomposition could allow the seeds to remain intact in the feces for longer periods without any damage. Since this study collected all available feces, there could be a possibility that seeds deposited in different seasons were also collected showing the patterns in the findings of this study. Future research on endozoochorous seed dispersal by raccoon dogs should investigate dispersal patterns across different life stages, seasons and under different environmental conditions within urban settings and examine the role of different urbanization indicators on urban‐adapted dispersers. Nevertheless, our findings suggest that while raccoon dogs serve as effective seed dispersers, urbanization may affect the habitat diversity facilities provided to endozoochorous seed dispersal services.

## 5 Conclusion

Research on the impact of urbanization has largely focused on species diversity (White et al. 2018), often overlooking its influence on ecological interactions such as endozoochorous seed dispersal. As urbanization expands rapidly, understanding the effects of habitat diversity on seed dispersal processes, such as endozoochory, is crucial for sustaining the ecological processes and services provided by animals (Yadav et al. 2025). For instance, habitat diversity at large scales may facilitate interactions between urban‐adapted dispersers and a broader range of plant species. However, when habitat diversity is analyzed alongside urbanization at small scales, even well‐adapted dispersers can potentially face challenges in animal‐mediated seed dispersal. This decline in seed‐dispersal efficiency can negatively affect plant diversity in novel habitats (McConkey and O'Farrill 2016). Given these findings, we recommend prioritizing ecological processes, such as endozoochorous seed dispersal, by urban‐adapted animals because they substantially contribute to urban greening. Future research should consider a broader range of urban indicators to gain a better understanding of habitat preferences among active dispersers, such as raccoon dogs. Strengthening knowledge in this area may provide opportunities for plant dispersal and establishment and promote biodiversity conservation in urban environments.

## Author Contributions

**Harsh Yadav:** conceptualization (equal), data curation (equal), formal analysis (equal), funding acquisition (equal), investigation (equal), methodology (equal), project administration (equal), resources (equal), software (equal), validation (equal), visualization (equal), writing – original draft (equal), writing – review and editing (equal). **Yuki Iwachido:** conceptualization (supporting), data curation (supporting), funding acquisition (equal), investigation (equal), methodology (supporting), project administration (supporting), resources (supporting), supervision (equal), validation (equal), writing – review and editing (equal). **Shyam S. Phartyal:** validation (equal), writing – review and editing (equal). **Takehiro Sasaki:** funding acquisition (equal), methodology (supporting), project administration (supporting), resources (equal), supervision (equal), validation (equal), writing – review and editing (equal).

## Ethics Statement

The authors have nothing to report.

## Conflicts of Interest

The authors declare no conflicts of interest.

## Supporting information


**Table S1:** ece372516‐sup‐0001‐TableS1.csv.

**Table S2:** ece372516‐sup‐0002‐Supinfo.docx.

## Data Availability

Data is available in Supporting Information.
