# Supplementary material for: Urbanization and Habitat Diversity Promote Endozoochorous Seed Dispersal by Raccoon Dogs Within Forest Fragments in Tokyo
Source: Ecol Evol. 2025 Nov 14;15(11):e72516. doi: 10.1002/ece3.72516 (PMC12616490; doi:10.1002/ece3.72516)

**Urbanization and habitat diversity promote endozoochorous seed dispersal by raccoon dogs within forest fragments in Tokyo**

**Harsh Yadav^1*^, Yuki Iwachido^1^, Shyam S. Phartyal^2^ and Takehiro Sasaki^1, 3^**

^1^Graduate School of Environment and Information Sciences, Yokohama National University, Yokohama, Kanagawa, 240-8501, Japan

^2^Department of Forestry, Mizoram University, Aizawl, 796004, India

^3^Institute for Multidisciplinary Sciences, Yokohama National University, Yokohama, Kanagawa, 240-8501, Japan

***Corresponding author: Harsh Yadav** ([harshyadav938@gmail.com](mailto:harshyadav938@gmail.com)) **and Takehiro Sasaki** ([sasaki-takehiro-kw@ynu.ac.jp](mailto:sasaki-takehiro-kw@ynu.ac.jp))

Table S2: Description of sites along with number of species and seeds documented in the feces of raccoon dogs.

| **Site** | **No. of toilets** | **No. of samples** | **No. of species** | **No. of seeds** | **No. of viable seeds** | **No. of damaged seeds** | **No. of native species** | **No. of native seeds** | **No. of non-native species** | **No. of non-native seeds** | **No. of plant species in the site** | **No. of common plant species (site vs feces)** |
| --- | --- | --- | --- | --- | --- | --- | --- | --- | --- | --- | --- | --- |
| Zama Yatoyama Park | 2 | 7 | 25 | 286 | 252 | 34 | 18 | 251 | 7 | 35 | 74 | 6 |
| Ikego Forest Natural Park | 3 | 8 | 19 | 129 | 101 | 28 | 16 | 123 | 3 | 6 | 61 | 8 |
| Miwa Midori Park | 3 | 5 | 18 | 156 | 137 | 19 | 13 | 136 | 5 | 20 | 74 | 5 |
| Yokohama National University | 2 | 8 | 16 | 494 | 446 | 42 | 14 | 488 | 2 | 6 | 41 | 7 |
| Shinbayashi Park | 1 | 3 | 15 | 181 | 147 | 28 | 12 | 177 | 3 | 4 | 35 | 9 |
| Sagamihara Chuo Green Space | 2 | 2 | 15 | 190 | 171 | 19 | 11 | 179 | 4 | 11 | 75 | 4 |
| Maioka Park | 1 | 3 | 14 | 220 | 195 | 25 | 11 | 161 | 3 | 59 | 48 | 5 |
| Komayama Park | 1 | 3 | 13 | 87 | 83 | 4 | 11 | 85 | 2 | 2 | 53 | 10 |
| Sakuragaoka Park | 2 | 3 | 13 | 46 | 44 | 2 | 11 | 43 | 2 | 3 | 55 | 8 |
| Hiromachi Ryokuchi Park | 1 | 2 | 13 | 78 | 71 | 10 | 10 | 74 | 3 | 4 | 39 | 9 |
| Kamakura Central Park | 1 | 4 | 11 | 132 | 120 | 12 | 11 | 132 | 0 | 0 | 71 | 9 |
| Nagaesakurayama Park | 1 | 2 | 11 | 76 | 67 | 9 | 7 | 71 | 4 | 5 | 37 | 6 |
| Tobiosan Park | 1 | 2 | 10 | 40 | 33 | 7 | 9 | 39 | 1 | 1 | 70 | 6 |
| Chigasaki Satoyama Park | 1 | 2 | 9 | 44 | 39 | 5 | 9 | 44 | 0 | 0 | 63 | 7 |
| Nagasaka Park | 4 | 5 | 9 | 117 | 109 | 8 | 8 | 101 | 1 | 16 | 47 | 2 |
| Ohira Park | 2 | 2 | 6 | 23 | 22 | 1 | 4 | 21 | 2 | 2 | 48 | 4 |
| Tsukuiko Shiroyama Park | 1 | 1 | 5 | 9 | 9 | 0 | 3 | 5 | 2 | 4 | 59 | 3 |
| Isehara Tonoyama Park | 1 | 2 | 2 | 34 | 33 | 1 | 2 | 34 | 0 | 0 | 75 | 1 |
| Oyamada Ryokuchi Park | 1 | 2 | 1 | 40 | 38 | 2 | 1 | 40 | 0 | 0 | 102 | 1 |

Table S3: Three urban indicators for each survey site.

| **Site** | **Built-up area (%)** | **Artificial light (w/cm^2^)** | **Population density (per km^2^)** |
| --- | --- | --- | --- |
| Zama Yatoyama Park | 70.25 | 20.5833 | 7596.94 |
| Ikego Forest Natural Park | 24.73 | 10.6444 | 3578.7 |
| Miwa Midori Park | 33.88 | 10.4722 | 7934.91 |
| Yokohama National University | 68.7 | 32.4889 | 8939.11 |
| Shinbayashi Park | 67.69 | 22.6294 | 6200.4 |
| Sagamihara Chuo Green Space | 59.41 | 20.1824 | 2332.32 |
| Maioka Park | 52.36 | 14.6 | 8939.11 |
| Komayama Park | 49.99 | 12.4632 | 3207.44 |
| Sakuragaoka Park | 42.09 | 16.2882 | 7623.28 |
| Hiromachi Ryokuchi Park | 51.75 | 12.3611 | 4699.25 |
| Kamakura Central Park | 50.17 | 14.2526 | 4656.82 |
| Nagaesakurayama Park | 48.7 | 13.3294 | 2376.18 |
| Tobiosan Park | 30.45 | 7.2611 | 2521.74 |
| Chigasaki Satoyama Park | 15.92 | 6.4235 | 6945.54 |
| Nagasaka Park | 3.9 | 2.4722 | 4366.15 |
| Ohira Park | 15.19 | 4.7944 | 5512.12 |
| Tsukuiko Shiroyama Park | 13.03 | 5.5529 | 2332.32 |
| Isehara Tonoyama Park | 14.64 | 5.85 | 1923.57 |
| Oyamada Ryokuchi Park | 15.355 | 7.25 | 6530.03 |

Table S4: Modeling results of three urban indicators with respect to plant species found in the feces.

| **Buffer** | **Urban indicator** | **Estimate** | **S. Error** | **p value** | **aic** |
| --- | --- | --- | --- | --- | --- |
| 200 | art light | 0.2968 | 0.0998 | 3E-03 | 177.5 |
| 400 | art light | 0.2967 | 0.1005 | 3E-03 | 177.5 |
| 600 | art light | 0.3114 | 0.0973 | 1E-03 | 176.5 |
| 800 | art light | 0.3153 | 0.0973 | 1E-03 | 176.3 |
| 1000 | art light | 0.3195 | 0.0041 | <0.001 | 176.1 |
| 1200 | art light | 0.3245 | 0.0041 | <0.001 | 175.8 |
| 1400 | art light | 0.3259 | 0.0040 | <0.001 | 175.7 |
| 1600 | art light | 0.3244 | 0.0041 | <0.001 | 175.9 |
| 1800 | art light | 0.3264 | 0.0041 | <0.001 | 175.8 |
| 2000 | art light | 0.3230 | 0.0988 | 1E-03 | 176.1 |
| 200 | built-up | 0.0098 | 0.0029 | 9E-04 | 183.5 |
| 400 | built-up | 0.0182 | 0.0026 | 5E-12 | 172.8 |
| 600 | built-up | 0.0193 | 0.0039 | 9E-07 | 165.5 |
| 800 | built-up | 0.0198 | 0.0035 | 2E-08 | 161 |
| 1000 | built-up | 0.0202 | 0.0033 | 1E-09 | 158.7 |
| 1200 | built-up | 0.0208 | 0.0033 | 2E-10 | 157.1 |
| 1400 | built-up | 0.0213 | 0.0034 | 5E-10 | 157.8 |
| 1600 | built-up | 0.0218 | 0.0036 | 2E-09 | 158.9 |
| 1800 | built-up | 0.0224 | 0.0038 | 3E-09 | 159.4 |
| 2000 | built-up | 0.0229 | 0.0039 | 6E-09 | 160.3 |
| 200 | pop dense | 0.1734 | 0.0034 | <0.001 | 183.3 |
| 400 | pop dense | 0.1737 | 0.0035 | <0.001 | 183.3 |
| 600 | pop dense | 0.1711 | 0.0034 | <0.001 | 183.3 |
| 800 | pop dense | 0.1673 | 0.0034 | <0.001 | 183.5 |
| 1000 | pop dense | 0.1630 | 0.0034 | <0.001 | 183.6 |
| 1200 | pop dense | 0.1599 | 0.0034 | <0.001 | 183.7 |
| 1400 | pop dense | 0.1578 | 0.0034 | <0.001 | 183.7 |
| 1600 | pop dense | 0.1548 | 0.0034 | <0.001 | 183.8 |
| 1800 | pop dense | 0.1524 | 0.0034 | <0.001 | 183.9 |
| 2000 | pop dense | 0.1511 | 0.0034 | <0.001 | 183.9 |

Figure S1: The zig-zag transect approach followed within the site to survey raccoon dogs feces. Green colour box represents the plant survey subplots within each main plot.


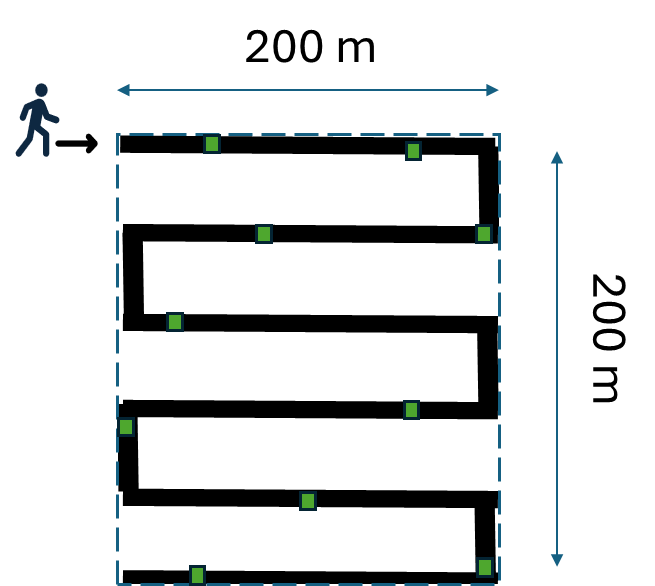


Figure S2: Number of native and non-native species found in the feces.


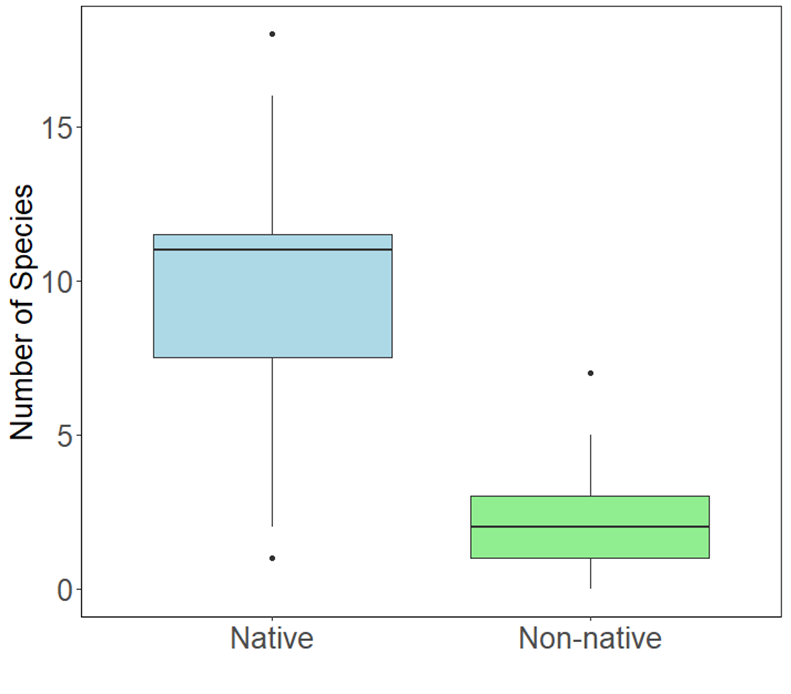


Figure S3: Number of viable and damages seeds found in the feces.


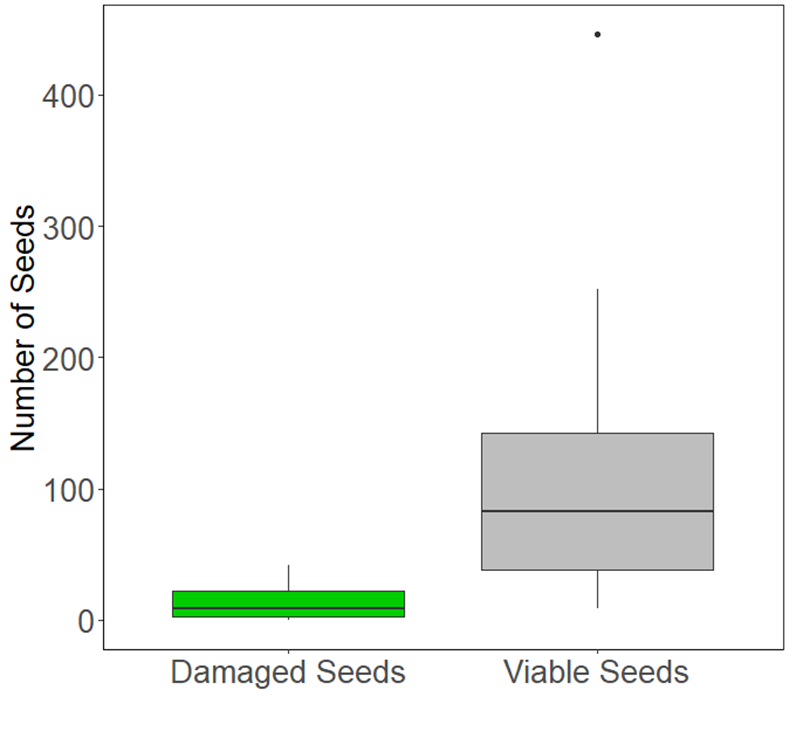


Fig. S4. Number of native and non-native species present in the feces and local vegetation at the respective sites. On the X axis, ‘Feces’ and ‘Site’ shows respective bars for native and non-native plant species.


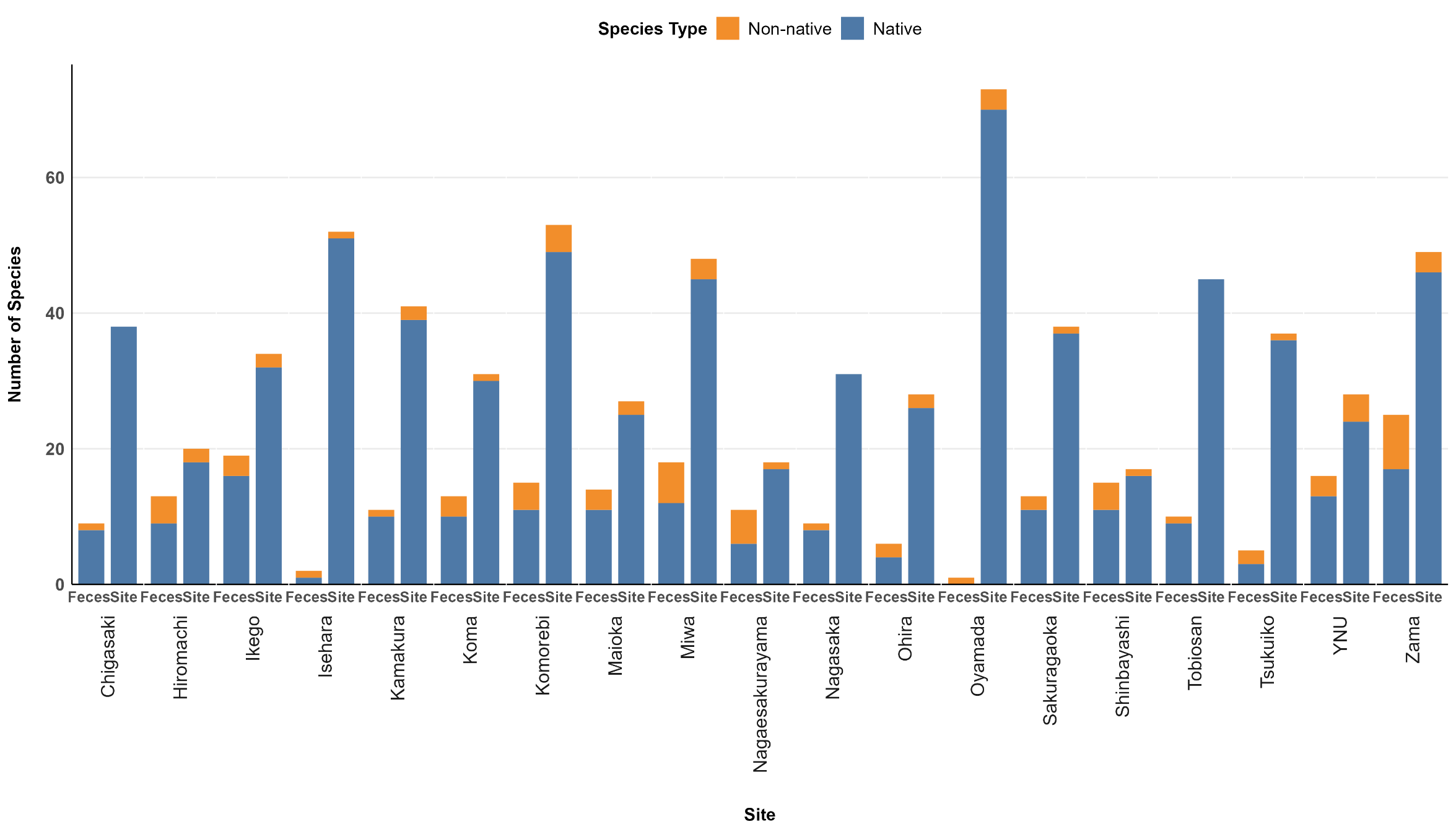


Fig. S5: Effects of habitat diversity and urbanization rate on number of seeds. The left side denotes the estimates of habitat diversity, while the right side indicates the estimates of urbanization rate within respective buffer radius categories. The colored points represent significant effects, whereas the tails indicate confidence intervals.


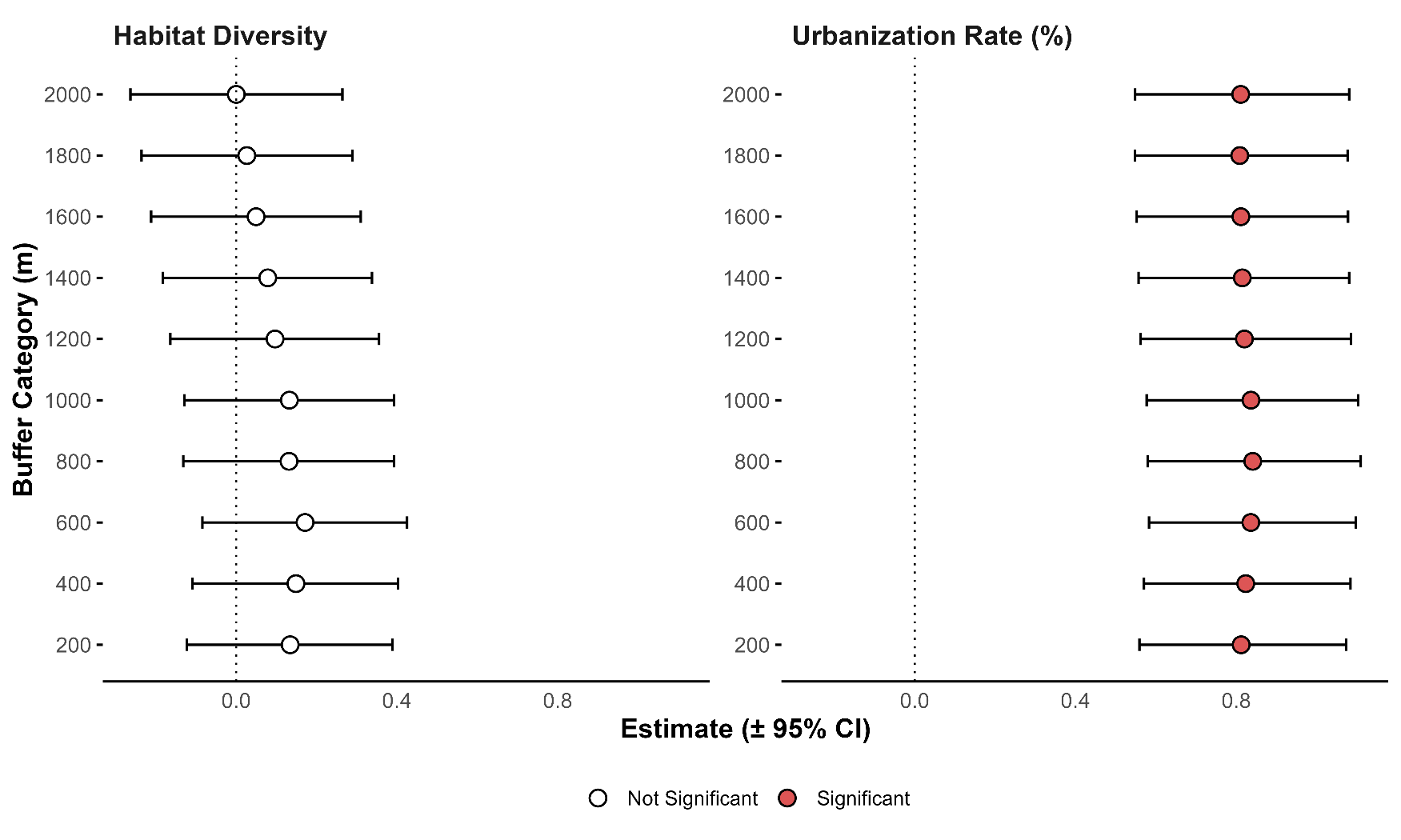

Supplement: Supplementary file 2 — Table S2: ece372516‐sup‐0002‐Supinfo.docx. [file ECE3-15-e72516-s001.docx]
